# Benchmark-Outcome Separation in Federal Independent Dispute Resolution: A Descriptive Analysis of Qualifying Payment Amount and Adjudicated Payment in Complex Repair Claims

**DOI:** 10.7759/cureus.110912

**Published:** 2026-06-15

**Authors:** Andrew M Klapper, Anthony N Dardano, Michael Risin, Karla Maita, Martha L Denavea

**Affiliations:** 1 Plastic and Reconstructive Surgery, Delray Medical Center, Delray Beach, USA; 2 Nursing/Revenue Cycle and Clinical Documentation, Floridian Institute of Plastic Surgery, Delray Beach, USA

**Keywords:** adjudicated rate index, complex repair, independent dispute resolution, no surprises act, out-of-network reimbursement, qualifying payment amount

## Abstract

Introduction

The Qualifying Payment Amount (QPA) is an issuer-calculated median in-network contracted rate defined by statute and regulation and is a central reference point in the federal No Surprises Act independent dispute resolution (IDR) process. Whether reported QPA behaves as a close descriptive comparator for observed federal IDR payment determinations in specific procedural domains remains an empirical question.

The objective of this study was to evaluate the relationship between reported QPA and observed adjudicated payment determinations in federal IDR disputes involving Current Procedural Terminology (CPT) 13100-13153 (complex repair and layered closure codes) and to introduce the Adjudicated Rate Index (ARI) as a descriptive cohort-level summary of adjudicated-payment-to-QPA multiples.

Methods

Centers for Medicare & Medicaid Services (CMS) federal IDR public use files were analyzed for disputes involving CPT 13100-13153. For each dispute, a case-level payment multiple was calculated as the selected payment divided by the reported QPA. ARI was defined as the median of these case-level multiples within a specified cohort. Prespecified analyses included all analytic cases, provider/facility-prevailing disputes, plan-prevailing disputes, quarter-level cohorts, and CPT subgroup cohorts. The analysis was descriptive and did not treat adjudicated payment as a normative payment standard, measure of clinical value, or predictor of individual dispute outcomes.

Results

Across prespecified analytic views, reported QPA and observed adjudicated payment determinations were not empirically interchangeable within this complex repair code family. In the pooled 2023 quarter 1 (Q1)-2025 quarter 2 (Q2) provider/facility-prevailing cohort, the median adjudicated-payment-to-QPA multiple was 28.73×. Across all analytic cases with available QPA-multiple data, the median multiple was 22.41×. Plan-prevailing disputes clustered near QPA parity, supporting the interpretation of the source field as reporting QPA-relative payment outcomes.

Conclusion

In CMS-reported federal IDR disputes involving CPT 13100-13153, reported QPA and observed adjudicated payment determinations did not behave as interchangeable descriptive reimbursement measures within the analyzed cohort. ARI provides a domain-specific summary of observed adjudicated-payment-to-QPA relationships, but it does not replace QPA in the statutory framework, establish payment adequacy, or predict individual dispute outcomes. Further analyses across other procedural domains, specialties, regions, payer types, and data sources are needed to assess generalizability.

## Introduction

The federal No Surprises Act (NSA) created an administrative pathway for resolving certain out-of-network payment disputes [1,2]. Within that framework, the Qualifying Payment Amount (QPA) serves as a central statutory and regulatory reference point. QPA is an issuer-calculated median in-network contracted rate. Whether reported QPA behaves as a close descriptive comparator for observed federal independent dispute resolution (IDR) payment determinations in specific procedural domains remains an empirical question. This study therefore asks a narrow descriptive question: within federal IDR disputes involving CPT 13100-13153, how closely do reported QPA values align with observed adjudicated payment determinations?

Surprise medical billing has been widely documented within emergency and out-of-network care settings where patients often have limited ability to select providers [3,4]. Prior analyses demonstrated substantial variability in out-of-network billing practices, reimbursement patterns, and stakeholder perceptions, contributing to broader federal policy intervention through the NSA [5-9]. More recent evaluations following NSA implementation have begun examining the relationship between administratively defined payment benchmarks and observed IDR outcomes, including discrepancies between QPA estimates and reimbursement determinations across selected specialties and dispute categories [10-12].

Prior work using the CASCADE (Cost Analysis of Surgical Complications and Downstream Expenditure) framework described how complex wound and reconstructive care may generate stage-dependent downstream expenditure trajectories that are not fully captured by isolated procedural payment amounts [13]. CASCADE was a conceptual cost-trajectory framework focused on total episode expenditure, including how delayed, inadequate, or failed reconstruction may shift costs across later stages of care. The present study does not test CASCADE directly. Rather, it extends the same general concern about construct separation to a narrower empirical question: whether an administrative benchmark and an adjudicated payment outcome behave as interchangeable reimbursement measures within a defined procedural domain. Specifically, it asks whether issuer-calculated administrative benchmarks and observed federal IDR payment determinations behave as close descriptive comparators within a defined complex repair code family, or whether they represent distinct reimbursement constructs.

Current Procedural Terminology (CPT) 13100-13153 was selected as a clinically coherent complex repair family that reduces heterogeneity within the public use file (PUF) while remaining relevant to reconstructive practice. The code family includes layered closure and complex repair services that are more internally comparable than a broader pooled surgical dataset. Restricting the analysis to this family improves internal comparability, although it narrows generalizability.

Federal IDR PUFs provide a reported dataset of completed disputes and corresponding QPA-relative payment outcomes [14]. Those files make it possible to examine, descriptively, how reimbursement sustained after IDR relates to QPA within a defined procedural cohort.

To summarize that relationship, this study introduces the Adjudicated Rate Index (ARI) as the cohort-level median of case-level adjudicated-payment-to-QPA multiples. ARI is a descriptive summary metric, not a payment rule, valuation method, legal standard, or replacement for QPA. The purpose of this analysis was to evaluate whether reported QPA functions as a close descriptive comparator for adjudicated reimbursement in this defined procedural domain and to provide a transparent cohort-level summary measure for that relationship.

## Materials and methods

Study objective

The primary objective was to evaluate the relationship between reported QPA and observed adjudicated payment determinations in federal IDR disputes involving CPT 13100-13153 across prespecified analytic cohorts. A secondary objective was to introduce ARI as a descriptive cohort-level summary of case-level adjudicated-payment-to-QPA multiples.

Data source

The data source was the Centers for Medicare & Medicaid Services (CMS) Federal IDR public use file for NSA disputes [14]. The analysis was limited to rows corresponding to CPT-coded disputes within the complex repair family 13100-13153. Because the CMS file is a national public use dataset, the analysis reflects national reported disputes rather than a balanced state-by-state sample. The study uses the CMS federal IDR PUF as published and does not independently validate insurer-reported QPA values, party-submitted offer amounts, certified IDR entity determinations, or source-field coding. The analysis was conducted using a public federal dataset, prespecified CPT family definitions, and transparent ratio-based calculations reproducible from the reported source fields. These limitations in source-field validation should be considered when interpreting any derived ARI values.

Rationale for code-family selection

CPT 13100-13153 was selected for clinical coherence, reduced heterogeneity within the public use file, and sufficient volume to support descriptive subgroup analyses. The code family was selected for methodological coherence rather than for prior knowledge of divergence magnitude.

Cohort construction

The primary descriptive analysis was the pooled 2023 quarter 1 (Q1)-2025 quarter 2 (Q2) all-case cohort with available QPA-multiple data. Prespecified subgroup analyses included provider/facility-prevailing disputes, plan-prevailing disputes, quarter-level cohorts, and CPT subgroup cohorts. The 2025 Q1 provider/facility-prevailing cohort was examined as a recent-quarter sensitivity analysis. These cohorts were not intended to estimate payment levels across all NSA claims, all federal IDR disputes, or broader commercial markets. The analytic cohort is not a random sample of all NSA-eligible claims, all out-of-network complex repair claims, or all disputes initiated through the federal IDR process. It is conditional on claims that entered IDR, were not withdrawn or settled before determination, reached a reported determination, and contained usable QPA-relative payment data. ARI should therefore be interpreted only as a descriptive statistic within the completed-IDR cohort and not as an estimate of reimbursement across all claims. Because CMS PUFs do not report the denominator of all potentially eligible complex repair claims, the degree of selection into IDR cannot be directly quantified from the source data. Selection is described within the observable completed-dispute dataset by separately reporting all analytic cases, provider/facility-prevailing disputes, plan-prevailing disputes, quarter-level cohorts, and CPT subgroup cohorts.

Exclusion Criteria

Rows were excluded if they did not correspond to CPT 13100-13153, lacked a valid reported QPA-relative payment field, or lacked a reported selected-payment outcome sufficient to calculate the case-level multiple. No imputation was performed. Disputes with unavailable or noninterpretable QPA-multiple data were excluded from ratio-based ARI calculations.

Definitions and metric construction

For purposes of this analysis, provider/facility-prevailing disputes are defined as disputes in which the certified IDR entity selected the provider's or facility's submitted offer; plan-prevailing disputes are defined as disputes in which the certified IDR entity selected the plan's submitted offer.

For each dispute i, a case-level adjudicated-payment-to-QPA multiple was calculated as Mᵢ = Awardᵢ/QPAᵢ, where Awardᵢ is the certified IDR entity payment determination, and QPAᵢ is the reported QPA for that dispute. ARI for a cohort was defined as the cohort-level median of Mᵢ across all disputes in that cohort: ARI(c, g, t) = Median(Mᵢ) for all i in cohort (c, g, t), where c is the CPT cohort, g is the geographic region, and t is the time period. ARI is therefore indexed to a defined cohort and should not be interpreted as a fixed constant applicable outside the analyzed dispute set. An ARI of 1.0 indicates that the cohort median certified payment equaled reported QPA; values above 1.0 indicate that the cohort median certified payment exceeded QPA by that multiple. ARI is a cohort-level median ratio, not a dollar-denominated payment standard.

The proportion of disputes with Mᵢ > 1.0 was computed as a secondary descriptive measure. The median was treated as the primary measure of central tendency because distributions of Mᵢ in IDR datasets are expected to be right-skewed and dispersed.

Stated more simply, ARI summarizes how many times higher or lower the median selected IDR payment was relative to reported QPA within a defined cohort. It is a ratio describing the relationship between two reported fields, not an independent valuation of the service.

Benchmark context

QPA is an issuer-calculated median in-network contracted rate defined under federal statute and regulation. ARI summarizes observed reimbursement outcomes after completed federal IDR within a defined cohort. The two measures originate from different processes and address different empirical questions; the analysis below evaluates how closely they align in this code family.

Analytic posture

This analysis is descriptive rather than normative. It does not propose a replacement for QPA, does not infer legislative or regulatory intent, and does not treat completed IDR outcomes as a correct payment standard. IDR-sustained reimbursement was treated as an observed outcome within a defined subset of disputes rather than a correct or preferred payment level. This study does not equate adjudicated payment determinations with clinical value, usual-and-customary payment, provider cost, billed charge, or an objectively correct reimbursement amount. The analysis addresses only whether reported QPA and observed federal IDR payment determinations behave as close descriptive comparators within a defined procedural cohort.

Key definitions and interpretive boundaries used throughout the analysis are summarized in Table 1.

**Table 1:** Key Definitions and Interpretive Boundaries ARI: Adjudicated Rate Index; QPA: Qualifying Payment Amount; IDR: Independent Dispute Resolution; NSA: No Surprises Act

Term	Definition	Interpretive boundary
QPA	Issuer-calculated median in-network contracted rate	Not clinical value, provider cost, or correct payment
Adjudicated payment	Certified IDR entity selected payment	Not fair value, usual-and-customary, or a market-wide estimate
ARI	Median adjudicated-payment-to-QPA multiple within a defined cohort	Not a payment rule, individual predictor, or payment standard
Benchmark-outcome separation	Observed divergence between administrative benchmark and adjudicated outcome	Not proof of payer intent, provider entitlement, or payment inadequacy
Completed-IDR cohort	Disputes reaching reported federal IDR outcome	Not all NSA-eligible claims; excludes settled, withdrawn, or unfiled disputes

Source-field interpretation and validation

The CMS PUF field labeled “Prevailing Party Offer as % of QPA” was interpreted as a QPA-relative selected-payment multiple rather than a literal percentage. To assess the internal consistency of this interpretation, a face-validity check was performed using plan-prevailing disputes. In the pooled plan-prevailing cohort across all 10 analyzed quarters, the median value was 1.00×, consistent with selected plan offers clustering near QPA parity. This internal pattern was observed in every individual quarter, including 2024 quarter 3 (Q3) (n=82; median=1.00×) and 2024 quarter 4 (Q4) (n=82; median=1.00×). This consistency supports, but does not independently prove, the interpretation of the field as a QPA-relative payment multiple. Because the public use file does not provide independent dollar-denominated selected payment and QPA values for each dispute, external validation of the field against source adjudication records was not possible. Any systematic CMS source-field coding or interpretation error would materially affect ARI values and is therefore treated as a material limitation.

Unit of analysis

The dispute was the underlying observational unit; ARI was summarized at the cohort level. Cohorts were defined by CPT code or prespecified CPT grouping and time period. Because the analysis was descriptive and the completed-IDR cohort was nonrandom, no inferential testing was used to establish causality, payment adequacy, external validity, or generalizability. All analyses were conducted in Python 3 using pandas, NumPy, and SciPy (Python Software Foundation, Wilmington, Delaware, United States).

CMS PUFs do not provide sufficient claim-line, batch-level, or party-level identifiers to fully decompose batched determinations into independent clinical encounters. Therefore, the dispute row was retained as the unit of analysis, and no batch-level clustering adjustment was performed. This may overweight disputes submitted in larger batches or by parties with high submission volumes. For this reason, ARI should be interpreted as a completed-dispute-level descriptive statistic rather than a patient-level, encounter-level, or market-level estimate. To partially address batching-related concentration, results were reported across CPT subgroup and quarter-level cohorts; however, these subgroup analyses do not eliminate potential within-batch or within-party correlation.

Statistical testing

Because the study was descriptive and the completed-IDR cohort was nonrandom, statistical testing was not used to establish causality, payment adequacy, market-wide reimbursement, or generalizability. A Wilcoxon signed-rank test comparing case-level multiples with QPA parity was included only as a distributional sensitivity check in supplementary data. The primary interpretation rests on descriptive medians, interquartile ranges (IQRs), prevailing-party stratification, CPT subgroup summaries, and quarter-level consistency across available data.

## Results

Across prespecified analytic views within CPT 13100-13153, reported QPA and observed adjudicated payment determinations were not empirically interchangeable within this code family. In the pooled all-case cohort (N = 4,276), the median adjudicated-payment-to-QPA multiple was 22.41×, and 91.0% of adjudicated payments exceeded the reported QPA. In the pooled 2023 Q1-2025 Q2 provider/facility-prevailing cohort (N = 3,638), the median multiple was 28.73×.

When plans prevailed (n = 635), the median multiple was 1.00×, consistent with selected plan offers approximating QPA parity in those cases and supporting the field interpretation. These multiples should be interpreted as QPA-relative outcomes within the reported completed-IDR cohort, not as estimates of commercial reimbursement levels across all complex repair claims. Distributional detail by cohort is presented in Table 2. 

**Table 2:** Adjudicated-Payment-to-QPA Multiples by Prevailing-Party Cohort (CPT 13100–13153; 2023 Q1–2025 Q2) ARI = cohort-level median of Mᵢ (selected payment/reported QPA). P25, P75, P90 = percentiles of Mᵢ distribution. %>QPA = proportion with Mᵢ > 1.0. Plan-prevailing P25/P75/P90 not shown: disputes cluster near QPA parity. N: number of disputes with available QPA-multiple data; ARI: Adjudicated Rate Index; QPA: Qualifying Payment Amount; CPT: Current Procedural Terminology; Q1: quarter 1; Q2: quarter 2

Cohort	N	Median ARI	P25	P75	P90	%>QPA
2025 Q1 provider/facility-prevailing (primary cohort)	679	29.13×	14.34×	55.22×	74.43×	98%
Provider/facility-prevailing, pooled 2023 Q1–2025 Q2	3638	28.73×	14.57×	51.42×	72.37×	99%
Plan-prevailing, pooled 2023 Q1–2025 Q2	635	1.00×	—	—	—	—
All analytic cases, 2023 Q1–2025 Q2	4276	22.41×	9.75×	47.31×	68.73×	91%

**Figure 1:**
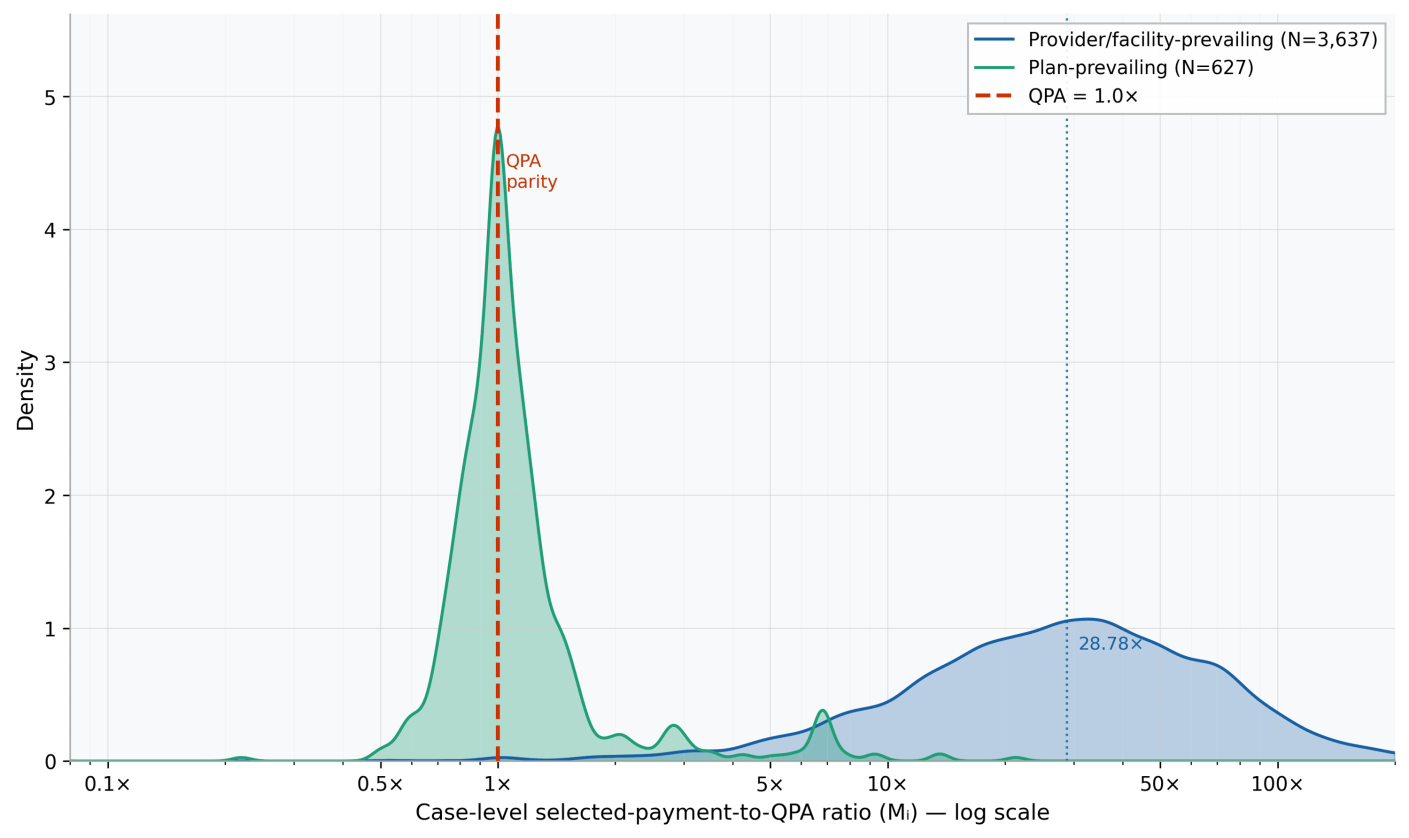
Distribution of Case-Level Selected-Payment-to-QPA Ratios by Prevailing-Party Result (CPT 13100–13153; 2023 Q1–2025 Q2; N = 4,276) Kernel density estimates of case-level Mᵢ values for provider/facility-prevailing (n = 3,638) and plan-prevailing (n = 635) disputes. Horizontal axis log-scaled. Dashed vertical lines indicate cohort medians. Vertical reference at 1.0× indicates QPA parity. The plan-prevailing distribution clusters near 1.0×, consistent with selected plan offers approximating QPA parity and supporting the QPA-multiple interpretation of the source field. Detailed cohort-level summary statistics for the distributions shown in Figure 1 are provided in Table 2. QPA: Qualifying Payment Amount; CPT: Current Procedural Terminology; Q1: quarter 1; ARI: Adjudicated Rate Index

Distributional analysis showed a right-skewed pattern of case-level adjudicated-payment-to-QPA multiples, with provider/facility-prevailing cases exhibiting substantially higher values compared to plan-prevailing cases, which clustered at QPA parity (Figure 1).

Across prespecified CPT subgroups, median adjudicated-payment-to-QPA multiples ranged from 12.69× for CPT 13101 to 26.02× for CPT 13131-13133, with all five CPT groupings showing median multiples materially above 1.0× and a majority of disputes exceeding QPA. Cohort-level detail is presented in Table 3. 

**Table 3:** Descriptive Summary Across Prespecified CPT Cohorts (CPT 13100–13153; All analytic cases; 2023 Q1–2025 Q2) ARI = cohort-level median of Mᵢ computed across all analytic disputes regardless of prevailing-party outcome. CPT 13100 (N < 5) grouped with 13101. Results conditional on disputes with available QPA multiples. QPA: Qualifying Payment Amount; CPT: Current Procedural Terminology; Q1: quarter 1; Q2: quarter 2

CPT	Anatomic site	N	Median ARI	P25	P75	P90	%>QPA
13101	Trunk/scalp, 2.6–7.5 cm	246	12.69×	5.14×	24.42×	46.17×	97%
13102	Trunk/scalp, ea addl 5 cm	257	21.87×	7.43×	53.26×	105.80×	95%
13120–13122	Scalp/axillae/genitalia	224	13.01×	5.14×	30.46×	59.43×	95%
13131–13133	Forehead/cheeks/chin	2064	26.02×	12.58×	50.01×	86.41×	98%
13150–13153	Eyelids/nose/ears/lips	1482	23.74×	11.28×	50.11×	83.01×	98%
All cohorts	Pooled	4276	22.41×	10.67×	48.03×	82.50×	97%

Across all 10 quarters (2023 Q1-2025 Q2, complete), median ARI values remained above QPA parity (1.0×) in every quarter, ranging from 11.32× (2023 Q1) to 26.50× (2024 Q4). The 2024 Q3 cohort (n = 567) produced a median ARI of 23.74× (P25 = 8.44×, P75 = 48.39×, %>QPA = 90.1%); the 2024 Q4 cohort (n = 811) produced a median ARI of 26.50× (P25 = 9.31×, P75 = 50.11×, %>QPA = 93.5%). Both quarters were consistent with all other analyzed quarters: ARI was materially above QPA parity, p < 0.001. Quarter-level detail is provided in the Appendices.

**Figure 2:**
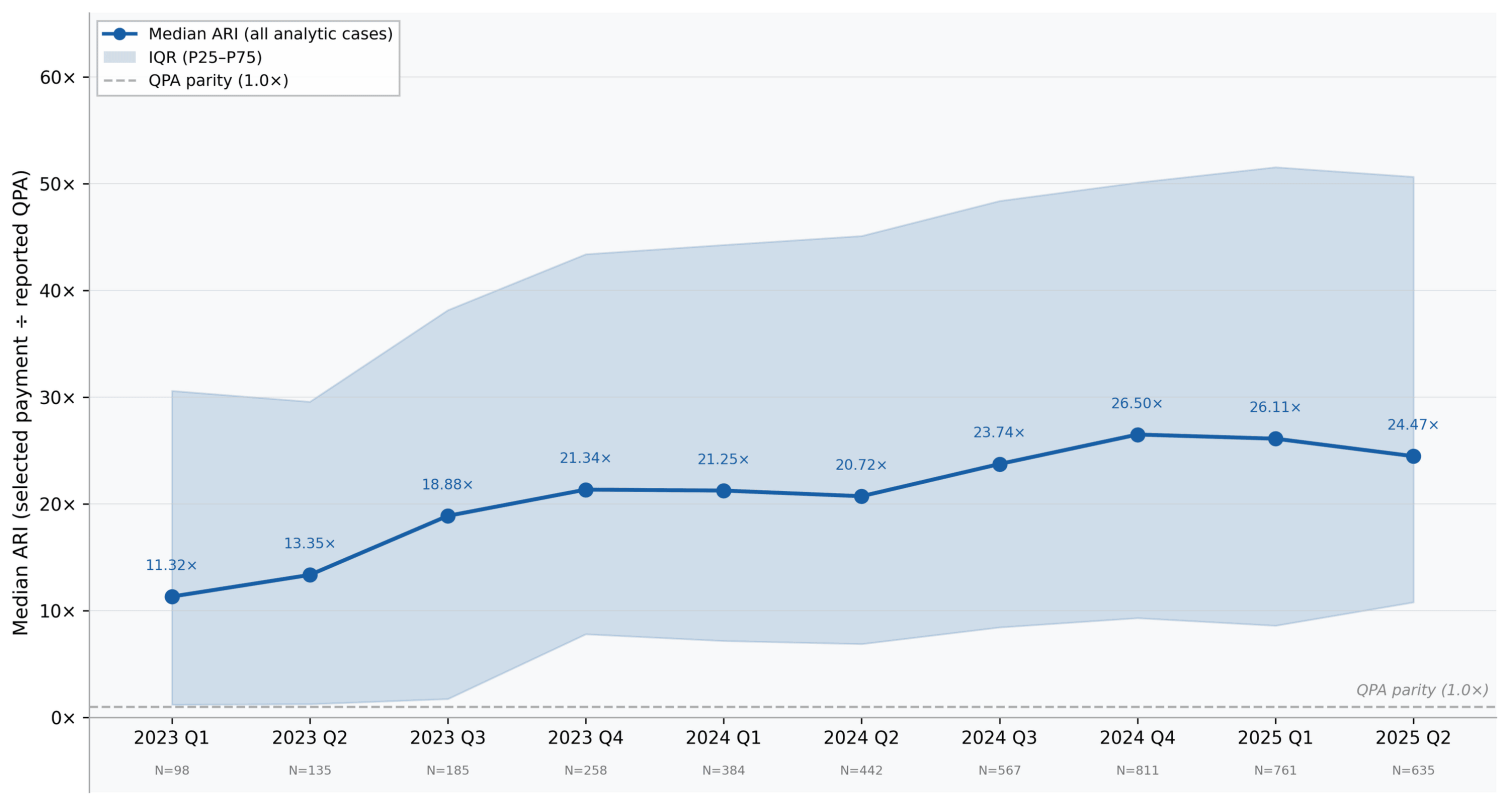
Quarter-Level ARI Across Study Period (CPT 13100–13153; all analytic cases; 2023 Q1-2025 Q2; N = 4,276) Quarter-level median ARI (M; = selected payment; ÷ reported QPA;) across all 10 quarters with available QPA-multiple data. Solid line = median ARI per quarter. Shaded band = IQR (P25-P75). Horizontal dotted line = QPA parity (ARI = 1.0x). 2024 Q3 (n = 567, ARI = 23.74x) and 2024 Q4 (n = 811, ARI = 26.50x) are now included; no quarter-level gap remains. Median ARI exceeded QPA parity in all 10 analyzed quarters. No trend line is drawn. ARI: Adjudicated Rate Index; QPA: Qualifying Payment Amount; IQR: interquartile range; CPT: Current Procedural Terminology

## Discussion

The central finding of this study is that reported QPA and observed adjudicated payment determinations did not behave as interchangeable descriptive reimbursement measures within this defined complex repair code family. Across prespecified analytic views, the two measures did not function as close descriptive comparators within this procedural cohort, a pattern consistent with benchmark-outcome separation [13].

These findings do not establish a correct payment level and should not be interpreted as a normative payment standard. Rather, they indicate that an issuer-calculated administrative benchmark and an observed adjudicated payment determination arise from different processes and may describe different economic constructs. The relevance of these findings is empirical comparability, not statutory interpretation and not payment adequacy in any individual dispute [1,2].

ARI is best understood as a descriptive cohort-level summary of observed adjudicated-payment-to-QPA relationships. It does not replace QPA in the statutory framework, predict outcomes in individual disputes, measure clinical value, or define usual-and-customary payment. ARI is not a new payment standard; it is a transparent median ratio summarizing a relationship already present in the public data. Its relevance depends on cohort definition, source-field reliability, procedural coherence, and appropriate interpretive limits. Used narrowly, it may help describe benchmark-outcome separation. Used broadly or normatively, it would exceed the design of this study [13].

A benchmark’s administrative availability does not establish its empirical interchangeability with observed payment outcomes in a specific procedural domain. This distinction is particularly relevant in complex procedural care, where coding, documentation, clinical heterogeneity, market structure, and case selection may influence the relationship between administrative benchmarks and observed payment determinations. Prior analyses following NSA implementation have similarly reported variation between QPA estimates and observed reimbursement outcomes across selected specialties and dispute categories [10-12]. The observed separation may reflect a combination of case selection, documentation quality, regional concentration, market structure, batching, payer behavior, provider behavior, specialty-specific factors, and other unmeasured variables. This study was not designed to apportion the contribution of those factors.

Several potential confounders should be considered when interpreting the observed separation. Completed IDR disputes may overrepresent claims in which the submitting party believed the QPA was meaningfully below the appropriate payment amount, creating selection effects that are not visible in the PUF. Provider/facility-prevailing cases may also differ systematically from plan-prevailing cases in documentation quality, clinical complexity, coding specificity, regional market structure, batching strategy, or party offer behavior. In addition, the PUF does not provide sufficient information to determine whether high multiples reflect unusually low QPA values, unusually high selected offers, case-mix differences, payer-specific contracting patterns, or other unmeasured factors. These issues do not negate the descriptive observation that the reported constructs diverged within the completed-dispute cohort, but they limit causal and normative interpretation.

The results should be read as evidence of benchmark-outcome separation within a reported adjudicated dataset, not as a decision tool for individual dispute strategy. The present analysis does not estimate the economic yield of filing behavior. It does not observe claims that never entered IDR, disputes that were settled before determination, or cases that were withdrawn, lapsed, or otherwise excluded from reported outcomes [11,12]. ARI is therefore conditional on the completed-dispute dataset and should not be extrapolated to all NSA-eligible claims.

Future work should evaluate whether similar benchmark-outcome separation is present across other CPT families, specialties, regions, payer types, and time periods. Additional validation would require more granular clinical, geographic, payer, contract, documentation, and dispute-level data than are available in the PUF.

Limitations

This study has several limitations. The CMS federal IDR PUF is a de-identified administrative dataset and does not contain granular clinical variables, operative complexity, modifier-level context, payer contract details, documentation quality, batching structure, or complete information sufficient to model all sources of case-mix variation. The analysis is limited to disputes that reached reported federal IDR outcomes and therefore does not capture claims that settled, lapsed, were withdrawn, were resolved before IDR, or were never filed.

The analytic cohort is not a random sample of all NSA-eligible claims. It is conditional on disputes that entered federal IDR, reached reported determination, and contained usable QPA-relative payment data. The degree of selection into IDR cannot be directly quantified from the CMS PUF because the file does not report the denominator of all potentially eligible claims. Selection bias is described within the observable dataset by separately reporting all analytic cases, prevailing-party subgroups, CPT subgroups, and quarter-level cohorts.

A further limitation is that the analysis depends on the interpretation of the CMS PUF field reporting the prevailing-party offer relative to QPA. The observed clustering of plan-prevailing disputes near QPA parity supports the interpretation used in this study, but the public file does not permit independent verification of every underlying QPA, offer, or certified IDR entity calculation. Any systematic reporting, coding, or interpretation error in this field would affect the derived ARI values.

ARI is conditional on disputes that entered and completed the federal IDR process. It may therefore reflect filing behavior, party selection, documentation patterns, regional concentration, specialty-specific practice patterns, batching, and prevailing-party outcomes. It should not be interpreted as a market-wide payment benchmark, a usual-and-customary payment estimate, a measure of provider cost, a billed-charge benchmark, or a direct estimate of reimbursement across all NSA-eligible claims.

The analysis does not equate adjudicated payment determinations with clinical value or objectively correct payment. It evaluates only whether reported QPA and observed federal IDR payment determinations behaved as close descriptive comparators within a defined procedural cohort. The observed adjudicated-payment-to-QPA relationships should not be interpreted as expected returns from filing, as evidence of payer intent, as evidence of provider entitlement, or as a basis for predicting the likely outcome of any individual dispute.

The focus on CPT 13100-13153 improves internal procedural coherence but limits generalizability. Other code families, specialties, geographic regions, and dispute types may show smaller, larger, or directionally different relationships between reported QPA and adjudicated payment determinations. Because provider/facility-prevailing subgroup analyses are conditional on prevailing-party status, those values should not be interpreted as estimates across all federal IDR determinations.

This study is descriptive rather than causal. It cannot determine why certified IDR entities selected particular offers or what proportion of the observed separation reflects clinical complexity, market conditions, documentation quality, bargaining behavior, regional variation, batching, or other unmeasured factors. The absence of clinical severity adjustment limits causal interpretation but does not eliminate the descriptive observation that the two reported constructs were materially separated within the completed-dispute cohort. Future work should test the generalizability of ARI across additional procedural domains and evaluate whether ARI-like summaries remain stable when linked to more granular clinical, geographic, payer, and dispute-level data.

CMS PUFs do not permit full batch-level adjustment. The dispute row was retained as the unit of analysis, and ARI should not be interpreted as a patient-level, encounter-level, or market-level estimate. CPT subgroup and quarter-level analyses partially describe consistency across observable subsets but do not eliminate potential batching or party-level correlation.

## Conclusions

Within CMS-reported federal IDR disputes involving CPT 13100-13153, reported QPA and observed adjudicated payment determinations did not behave as interchangeable descriptive reimbursement measures within the analyzed completed-dispute cohort. ARI, defined as the cohort-level median of case-level adjudicated-payment-to-QPA multiples, provides a domain-specific descriptive summary of benchmark-outcome separation.

ARI does not replace QPA in the statutory framework, establish payment adequacy, measure clinical value, define usual-and-customary reimbursement, or predict individual dispute outcomes. Its utility is descriptive: it summarizes how adjudicated payment determinations relate to reported QPA within a defined procedural domain. Further analyses across other specialties, code families, geographic regions, payer types, and data sources are needed to determine whether similar benchmark-outcome separation is present beyond complex repair claims.

## Appendices

**Table 4:** Quarter-Level ARI Summary CPT 13100–13153 (All Analytic Cases; 10 Quarters With Available QPA Data) ARI = cohort-level median of Mᵢ within each quarter. Wilcoxon signed-rank tests against reference 1.0 (QPA parity), included as a distributional sensitivity check only. ARI: Adjudicated Rate Index; QPA: Qualifying Payment Amount

Quarter	N	Median ARI	P25	P75	P90	%>QPA	p	Note
2023 Q1	98	11.32×	1.20×	30.60×	42.41×	77%	<0.001	
2023 Q2	135	13.35×	1.25×	29.58×	54.18×	79%	<0.001	
2023 Q3	185	18.88×	1.73×	38.16×	57.89×	80%	<0.001	
2023 Q4	258	21.34×	7.80×	43.40×	60.57×	87%	<0.001	
2024 Q1	384	21.25×	7.17×	44.27×	65.00×	93%	<0.001	
2024 Q2	442	20.72×	6.88×	45.11×	72.29×	87%	<0.001	
2024 Q3	567	23.74x	8.44x	48.39x	74.48x	90%	<0.001	
2024 Q4	811	26.50x	9.31x	50.11x	75.23x	94%	<0.001	
2025 Q1	761	26.11×	8.60×	51.55×	72.98×	92%	<0.001	Primary cohort quarter
2025 Q2	635	24.47×	10.79×	50.65×	74.83×	92%	<0.001	
ALL	4,276	22.41×	10.67×	48.03×	82.50×	97%	<0.001	10 quarters; 0 excluded
